# Gut community alterations associated with *Clostridioides difficile* colonization in hospitalized gastroenterological patients with or without inflammatory bowel disease

**DOI:** 10.3389/fmicb.2022.988426

**Published:** 2022-09-06

**Authors:** Aleksander Mahnic, Spela Pintar, Pavel Skok, Maja Rupnik

**Affiliations:** ^1^National Laboratory for Health, Environment and Food, Department for Microbiological Research, Maribor, Slovenia; ^2^Department of Gastroenterology, University Medical Centre Ljubljana, Ljubljana, Slovenia; ^3^Faculty of Medicine, University of Maribor, Maribor, Slovenia; ^4^Department of Gastroenterology, University Clinical Centre Maribor, Maribor, Slovenia; ^5^Department of Microbiology, Faculty of Medicine, University of Maribor, Maribor, Slovenia

**Keywords:** inflammatory bowel disease, gut microbiota, *Enterococcus*, 16S amplicon sequencing, *Clostridium difficile*

## Abstract

*Clostridioides difficile* colonization and development of infection commonly occur in inflammatory bowel disease (IBD) patients and can trigger flare-ups. Both conditions are inherently linked to disrupted gut microbiota. This study included 149 hospitalized gastrointestinal patients, which were divided into IBD (*n* = 48) and non-IBD patients (*n* = 101). Patients were tested for *C. difficile* colonization (qPCR and selective plating), and gut bacterial communities were analyzed with 16S amplicon sequencing. Blood test results were retrospectively collected from the medical records. IBD and non-IBD patients had comparable *C. difficile* colonization rates (31.7 and 33.3%, respectively). Compared to non-IBD *C. difficile-*non-colonized patients, IBD and *C. difficile-*colonized patients shared multiple common bacterial community characteristics including decreased diversity and reduced abundance of strict anaerobic bacteria. Furthermore, certain microbiota alterations were enhanced when IBD was accompanied by *C. difficile* colonization, indicating a synergistic effect between both medical complications. Conversely, certain microbial patterns were specific to *C. difficile* colonization, e.g., co-occurrence with *Enterococcus*, which was most common in IBD patients (81.3%).

## Introduction

Inflammatory bowel disease (IBD) is a chronic complication in the gastrointestinal tract that is increasing in incidence worldwide (Kaplan, 2015; Alatab et al., 2020; Freeman et al., 2021). The two principal types of IBD, Crohn’s disease and ulcerative colitis, are distinguished based on the localization of inflammation along the gastrointestinal tract (Guan, 2019). IBD flare-ups are caused by activated immune responses against native intestinal microbiota in genetically predisposed individuals and can be triggered by a variety of environmental factors (Zhao and Burisch, 2019). One important risk factor for flare-ups is a preceding gastrointestinal tract infection. The most common infection that leads to flare-ups is caused by *Clostridioides difficile* (Khanna et al., 2017), whereas other bacterial pathogens are less common in this regard (Hanada et al., 2018). The colonization rate with *C. difficile* as well as the incidence of *C. difficile* infection (CDI) are higher in IBD patients, especially in patients with ulcerative colitis (Rodemann et al., 2007; Clayton et al., 2009; Tai et al., 2021). CDI manifestations are also more severe in IBD patients, more likely to lead to recurrent infections, and more often require colectomy and other gastrointestinal tract surgeries (Ananthakrishnan et al., 2008; Khanna and Pardi, 2016; Razik et al., 2016). Therefore, a better understanding of concomitant CDI and IBD could help improve treatment practices, such as fecal microbiota transplantation (Khoruts et al., 2016).

The evidence regarding gut community alterations in IBD patients is substantial. The most prominent alterations include decreased diversity of commensal communities, mainly Bacillota (synonym Firmicutes), and the consequent overgrowth of facultative anaerobes, often opportunistic pathogens such as enterobacteria (Khan et al., 2019; Qiu et al., 2022). Similar patterns are characteristic of the gut microbiota in CDI, in addition to *C. difficile* itself often becoming the predominant species in the gut (Sehgal and Khanna, 2021; Schnizlein and Young, 2022). Alterations in human gut microbiota in response to *C. difficile* colonization are in contrast poorly described (Zhang et al., 2015; Vincent et al., 2016). But it is well established that the commensal microbiota plays a crucial role at providing colonization resistance against *C. difficile* as demonstrated both *in vitro* and *in vivo* (Auchtung et al., 2020; Pike and Theriot, 2020).

Although *C. difficile* colonization is common in IBD patients, this study is the first to compare *C. difficile* colonization-associated alterations in the gut microbiota of IBD and non-IBD gastrointestinal patients. We used selective culturing and species-specific qPCR to detect and quantify *C. difficile* in stool samples from 149 hospitalized patients. Additionally, we performed 16S amplicon metagenomic sequencing to determine *C. difficile* colonization-associated bacterial community characteristics in IBD versus non-IBD patients.

## Materials and methods

### Sample collection

Stool samples were collected from 149 patients hospitalized at the Department of Gastroenterology, University Medical Centre Maribor, after informed consent. The same cohort was included in a broader study that compared microbiota signatures between healthy controls and hospitalized gastrointestinal patients (Mahnic et al., 2020). Patients were diagnosed with standard medical procedures including clinical, radiological, endoscopic, and histological criteria and were, for the purpose of further analysis, distributed into two groups based on the diagnosis: IBD (*n* = 48) and non-IBD patients (*n* = 101). The IBD group included patients with both principal IBD types, i.e., ulcerative colitis (*n* = 28) and Crohn’s disease (*n* = 20). The non-IBD group comprised patients with the following pathologies: infections (pneumonia, cholangitis, hepatitis, gastritis, or pancreatitis), cancer (pancreatic, gastric, or liver), liver cirrhosis, and peptic ulcer. Because patients were hospitalized for different medical complication and subsequently underwent different treatments, no exclusion criteria except minimum age of 18 was used in this study. All patient’s collected metadata can be found in Supplementary Table 1. No patient was hospitalized primarily due to CDI; however, three IBD patients tested positive for *C. difficile* during hospitalization. The cohort consisted of 76 women and 73 men, with an average age of 59.3 years (SD = 17.9). Values for C-reactive protein, leukocytes, neutrophil granulocytes, sedimentation rate, albumins, and ferritin were retrospectively collected from the medical records, and the test result closest to the sample collection date was taken for each patient. Ethical approval was obtained from the National Medical Ethics Committee (KME 95/05/15).

Stool samples were collected in sterile containers and immediately transported to the laboratory. After homogenization, a volumetric equivalent of 50 μL was added to 1 mL of Inhibitex buffer (QIAamp Fast Stool DNA Mini Kit, Qiagen, Hilden, Germany) and stored at −80°C until total DNA isolation.

### Total DNA isolation

After mechanical disruption (7,000 rpm for 70 s) with the SeptiFast Lyse Kit and MagNA Lyser (Roche, Basel, Switzerland), total bacterial DNA was extracted from each stool sample using the QIAamp Fast Stool DNA Mini Kit (Qiagen, Hilden, Germany) and used for molecular detection of *C. difficile* and microbiota analysis.

### Quantification of *Clostridioides difficile* 16S rRNA and *tcdB* genes

Both *C. difficile* 16S rRNA and *tcdB* genes were quantified with gene-specific qPCR using Rotor-Gene (Qiagen, Hilden, Germany) and Rotor-Gene Probe Mix (Qiagen, Hilden, Germany). For the amplification and detection of the 16S rRNA gene, we used F–(5′-TTGAGCGATTTACTTCGGTAAAGA-3′), R–(5′-CCATCCT GTACTGGCTCACCT-3′), Probe–(5′-CCTACCCTGTACACA CGGATAACATACCG-3′) (Rinttilä et al., 2004). For the amplification and detection of *tcdB*, we used F–(5′-GAAAGTCCAAGTTTACGCTCAAT-3′), R–(5′-GCTG CACCTAAACTTACACCA-3′), Probe–(5′-ACAGATGCAGC CAAAGTTGTTGAATT-3′) (van den Berg et al., 2006). qPCRs were performed in duplicate, and samples were considered positive for 16S rRNA or *tcdB* when both replicates were positive.

### *Clostridioides difficile* cultivation

Stool samples were cultivated by spreading a full inoculation loop on media selective for *C. difficile* (CHROMID; BioMerieux, Marcy-l’Etoile, France). This was followed by a 3-day incubation at 37°C under anaerobic conditions.

### 16S metagenomic sequencing library preparation and sequence data analysis

Bacterial community composition was obtained by paired-end sequencing of the V3V4 hypervariable region of the 16S rRNA gene on the MiSeq platform (Illumina, San Diego, CA, United States), as previously described (Mahnic et al., 2020).

Quality filtering of sequence reads and clustering into operational taxonomic units (OTUs) based on 97% similarity was performed using mothur (v.1.44.1) (Schloss et al., 2009), as previously described (Mahnic et al., 2020). Taxonomic classification of representative sequences was inferred using the RDP mothur training set v16. Down-stream statistical analyses and data visualization were performed in R (version 3.1.3) using package “ggplot2” and mothur (Shannon index, AMOVA, LEfSe).

The sequence data supporting the conclusion of this article are available in the form of combined paired-end reads (contigs) on the Metagenomics RAST (MG-RAST) database server under the project access number mgp86691. Samples used in this study are denoted with sample names G[sequential number of the sample].

## Results

In total, 48 IBD and 101 non-IBD patients were tested for colonization with *C. difficile* using a molecular approach (qPCR) and selective plating (Table 1). For statistical analysis, the IBD group was not further sub-divided into Crohn’s patients and those with ulcerative colitis because of the limited number of samples available. The study cohort was well balanced regarding gender; however, the mean age was significantly higher in non-IBD compared to IBD patients (*p* < 0.001) (Table 1). Age discrepancy was not compensated for in the statistical analysis; however, all reported results were critically evaluated with respect to this imbalance. Age related alterations in community structure are described in Supplementary Figure 1.

**TABLE 1:** Study cohort characteristics and *Clostridioides difficile* testing.

			qPCR		Culture
	Gender	Age	*C. difficile*	*tcdB*	
	[Male/Female]	[mean ± SD]	*N* (%)	*N* (%)	*N* (%)
Non-IBD (*n* = 101)	50/51	66.1 ± 14.6	32 (31.7%)	4 (4.0%)	3 (3.0%)
IBD (*n* = 48)	23/25	45.1 ± 15.5	16 (33.3%)	3 (6.3%)	2 (4.2%)

IBD: inflammatory bowel disease.

Using *C. difficile* species-specific qPCR, we detected *C. difficile* in 48 (32.2%) stool samples. We did not observe differences in the qPCR-based prevalence of *C. difficile* (Fisher’s exact test *p* = 0.853) or *tcdB* (Fisher’s exact test *p* = 0.681) or culture-based prevalence of *C. difficile* (Fisher’s exact test *p* = 0.657) between IBD and non-IBD patients (Table 1). All *tcdB*-positive and culture-positive samples were also detected with species-specific *C. difficile* qPCR (Supplementary Table 1). Hereinafter, all *C. difficile* qPCR positive samples were considered as *C. difficile-*colonized. Cultured strains belonged to four different *C. difficile* ribotypes: 001/072, 027, SLO 076, and 002. Out of 5 culture positive samples we were able to detect *tcdB in* 3 samples (60%). Samples that were negative after selective plating were additionally enriched prior to plating in brain heart infusion medium with 0.1% L-cysteine, 0.5% yeast extract, and 0.1% sodium taurocholate. After 5 days of incubation at 37°C under anaerobic conditions, no additional *C. difficile-*positive samples were obtained compared to direct plating on selective media (data not shown).

Furthermore, no correlations were observed between *C. difficile* colonization and the following blood markers: inflammatory markers (C-reactive protein, leukocytes, neutrophil granulocytes, and sedimentation rate), albumins, and ferritin (Supplementary Figure 2).

### *Clostridioides difficile* colonization-associated bacterial community characteristics are significant only in non-inflammatory bowel disease patients

**FIGURE 1:**
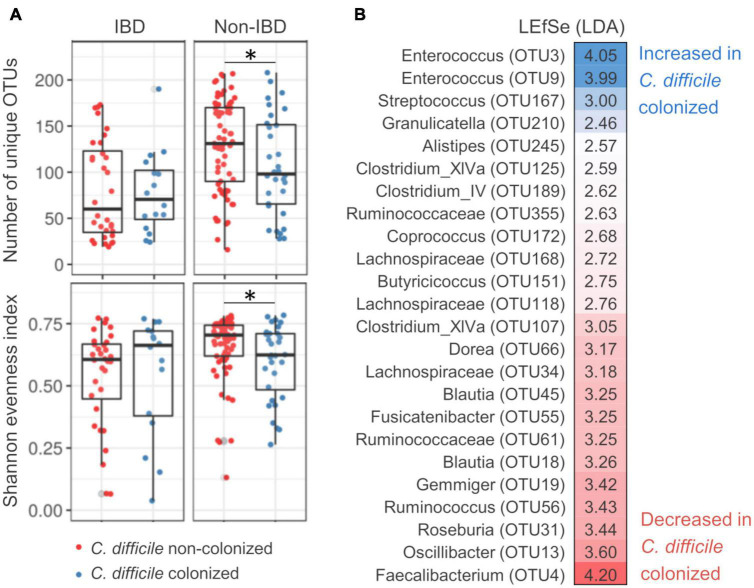
Bacterial community characteristics of *Clostridioides difficile-*colonized patients with regard to inflammatory bowel disease status. **(A)** *Clostridioides difficile* colonization-associated differences in bacterial community richness [number of unique operational taxonomic units (OTUs), top] and evenness (Shannon evenness, bottom) for inflammatory bowel disease (IBD) and non-IBD patients. **(B)** OTUs that were differentially represented between *C. difficile-*colonized and non-colonized patients, as obtained by LEfSe population analysis. Population analysis was performed only on non-IBD patients for which beta-diversity analysis showed significant differentiation between *C. difficile-*colonized and non-colonized. *Denotes significance with *p* value between 0.05 and 0.01.

Permutational multivariate analysis of variance (PERMANOVA) showed significant correlations of bacterial community structure with age, IBD status, and *C. difficile* colonization, explaining 1.5, 1.1, and 1.2% of inter-sample variability, respectively (Supplementary Table 2). Age-associated community characteristics are presented in Supplementary Figure 1. They did not correlate with differences reported in the context of IBD status or *C. difficile* colonization and will therefore not be discussed further. Differences in bacterial community structure between *C. difficile-*colonized and non-colonized patients were significant in non-IBD (AMOVA, *p* = 0.018) but not IBD patients (AMOVA, *p* = 0.967). In non-IBD patients, *C. difficile* colonization was associated with lower bacterial community richness (i.e., number of unique OTUs, *p* = 0.038) and community evenness (i.e., Shannon evenness, *p* = 0.045) (Figure 1A). Differentially represented OTUs included increased facultative anaerobic genera, e.g., *Enterococcus* and *Streptococcus*, and decreased strictly anaerobic genera, e.g., *Faecalibacterium, Roseburia, Blautia, Lachnospiraceae*, and *Clostridium* XIVa (Figure 1B).

The genus *Enterococcus* was significantly increased in *C. difficile-*colonized patients (Figure 1B). In our dataset, *Enterococcus* clustered into two distinct OTUs (OTU3 and OTU9). Both *Enterococcus*-associated OTUs exhibited a higher prevalence in *C. difficile*-colonized IBD and non-IBD patients [Fisher’s exact test: *p* = 0.004 (OTU3) and *p* = 0.020 (OTU9)]. The highest prevalence of *Enterococcus* was detected in *C. difficile-*colonized IBD patients (78.1, 56.3, and 81.3% for OTU3, OTU9, and both OTUs combined, respectively). Additionally, co-occurrence of both *Enterococcus*-associated OTUs was more frequent in *C. difficile*-colonized compared to non-colonized patients (Fisher’s exact test, *p* = 0.006).

### *Clostridioides difficile* colonization and inflammatory bowel disease show similar bacterial community alterations

**FIGURE 2:**
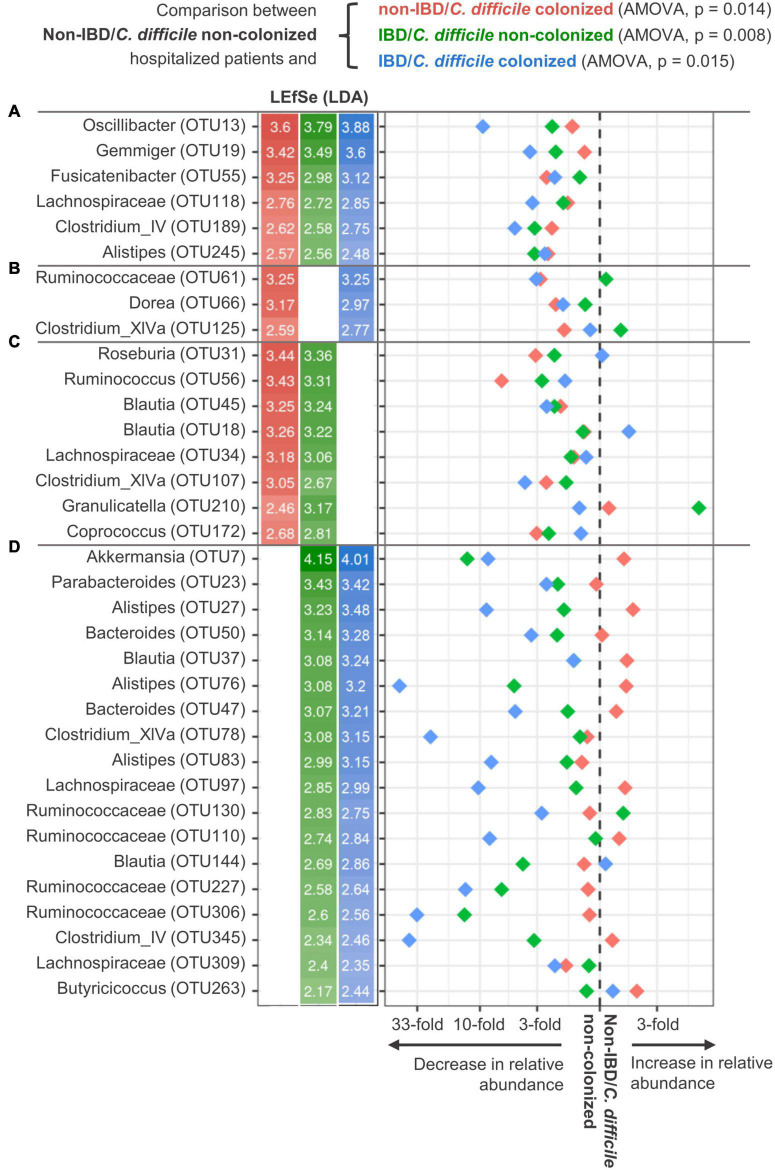
Shared community characteristics of *Clostridioides difficile* colonization and inflammatory bowel disease. Four groups were formed based on inflammatory bowel disease (IBD) status and *C. difficile* colonization. The control group contained *C. difficile* non-colonized non-IBD patients. For each differentially represented operational taxonomic unit (OTU), the LDA score (LEfSe test, left) and difference in relative abundance (right) for non-IBD/*C. difficile*-colonized (red), IBD/*C. difficile-*non-colonized (green), and IBD/*C. difficile*-colonized patients (blue) as compared to the control group are presented. Sections **(A–D)** denote OTU groups that are shared among the different patient groups.

Inflammatory bowel disease patients showed similar community characteristics as described above for *C. difficile*-colonized patients, partially explaining the lack of differentiation between *C. difficile-*colonized and non-colonized IBD patients. These characteristics included reduced community richness (*p* < 0.001) and a decrease in relative abundance of multiple Bacillota representatives. We demonstrated that multiple differentially represented OTUs were characteristic of both conditions, the underlying IBD as well as *C. difficile* colonization (Figures 2A,C). From the 24 OTUs associated with *C. difficile* colonization (Figure 1B), 14 (58.3%) were also associated with IBD. Three OTUs were significantly decreased in *C. difficile*-colonized in both the IBD and non-IBD group, but not in *C. difficile* non-colonized patients, *Ruminococcaceae* (OTU61), *Dorea* (OTU66), and *Clostridium* XIVa (OTU125) (Figure 2B). Interestingly, a few OTUs that were decreased in IBD patients showed significantly more prominent reduction when IBD was accompanied with *C. difficile* colonization, indicating potential synergistic effects of these two medical conditions (Figure 2D). These OTUs included multiple representatives of *Alistipes, Ruminococcaceae*, *Clostridium* XIVa, *Clostridium* IV, and *Lachnospiraceae* (Figure 2D).

## Discussion

Gut microbiota perturbations can be a precondition as well as a consequence of both IBD and CDI (Missaghi et al., 2014; Guan, 2019; Sehgal and Khanna, 2021; Qiu et al., 2022). While co-occurrence of IBD and CDI is common (Khanna et al., 2017), this study demonstrated that these two medical conditions also share common gut community signatures.

We did not observe a higher *C. difficile* colonization rate in IBD compared to non-IBD patients, despite sound evidence in the literature supporting this phenomenon. Studies not only report higher prevalence of *C. difficile* colonization in the population of IBD compared to non-IBD subjects (Bossuyt et al., 2009; Hourigan et al., 2013; Ramos-Martínez et al., 2015) but also the increase in prevalence with time (Issa et al., 2007; Mabardy et al., 2017). We also observed no correlation between *C. difficile* colonization and inflammatory markers in the blood, even though several inflammatory markers have been reported as predictive for adverse outcomes in cases of symptomatic CDIs (Dieterle et al., 2020). The absence of comparable conclusion in our study could be due to the small sample size, which we consider the main limitation of this study. Additionally, our study included only hospitalized gastroenterological patients, while comparable studies often include also healthy subjects as a control group.

The microbial community characteristics associated with *C. difficile* colonization in this study are in agreement with previous findings in asymptomatic carriers (Zhang et al., 2015) and CDI patients. Studies on CDI patients in concordance with this study reported decreased community diversity (Zhang et al., 2015; Milani et al., 2016; Duan et al., 2020) and a blooming effect in the gut, generally characterized by overgrowth of facultative anaerobic opportunists at the expense of strictly anaerobic commensals (Schubert et al., 2014; Milani et al., 2016; Amrane et al., 2019; Duan et al., 2020). Our findings also support the previously reported common co-occurrence of *C. difficile* and *Enterococcus* (Poduval et al., 2000; Fujitani et al., 2011), which was the most frequent genus (81.3%) in IBD patients in our study. Furthermore, the reduced abundance of *Ruminococcaceae*, *Dorea*, and *Clostridium* XIVa was exclusively associated with *C. difficile* colonization, regardless of IBD status. However, further testing is required to verify potential antagonistic effects.

Many of the above-mentioned *C. difficile*-associated microbial alterations are also common in IBD (Ni et al., 2017; Khan et al., 2019; Lloyd-Price et al., 2019), as also revealed by our current study. The driver of such changes in the microbiome is most likely the increased level of oxygen in the gut during inflammation, which occurs through different mechanisms (Rigottier-Gois, 2013; Rivera-Chávez et al., 2017). Nevertheless, an interesting aspect of our findings is the potential synergistic effect of concomitant IBD and CDI. In IBD patients, several taxa were markedly more decreased in *C. difficile*-colonized compared to non-colonized patients, including three OTUs associated with *Alistipes*. The genus *Alistipes* was previously reported to be negatively correlated with CDI both in humans (Milani et al., 2016) and in a murine model (Schubert et al., 2015), whereas its role in other medical complications remains under investigation (Parker et al., 2020). Synergistic effects in gut microbiota could potentially elucidate the mechanisms underlying more severe CDIs in IBD patients (Ananthakrishnan et al., 2008; Khanna and Pardi, 2016; Razik et al., 2016).

In conclusion, our comparison of *C. difficile-*associated patterns in the microbiome of hospitalized IBD and non-IBD patients has contributed to a better differentiation between disease-specific patterns and other alterations caused by external factors. We observed common characteristics of “gut blooming,” including reduced bacterial richness due to a decreased abundance of multiple Bacillota and overgrowth of facultative anaerobes. These characteristics were observed in IBD patients regardless of *C. difficile* status and in *C. difficile-*colonized patients regardless of IBD status. Importantly, synergistic effects between both medical conditions were detected, potentially explaining the more severe pathologies observed in concomitant IBD/CDI patients.

## Data availability statement

The datasets presented in this study can be found in online repositories. The names of the repository/repositories and accession number(s) can be found below: https://www.mg-rast.org/mgmain.html?mgpage=project&project=mgp86691, mgp86691.

## Ethics statement

The studies involving human participants were reviewed and approved by National Medical Ethics Committee (KME 95/05/15). The patients/participants provided their written informed consent to participate in this study.

## Author contributions

AM performed sequencing and data analysis and was a major contributor in writing the manuscript. SP partially performed total DNA isolations, retrieved and organized clinical data, and performed qPCR tests. PS organized clinical study and sample collection. MR contributed to project design, operation, and data interpretation and was a major contributor in writing the manuscript. All authors contributed to the article and approved the submitted version.
