# Supplementary material for: Gut community alterations associated with Clostridioides difficile colonization in hospitalized gastroenterological patients with or without inflammatory bowel disease
Source: Front Microbiol. 2022 Sep 6;13:988426. doi: 10.3389/fmicb.2022.988426 (PMC9485611; doi:10.3389/fmicb.2022.988426)
Supplement: Supplementary file 1 [file Data_Sheet_1.PDF]

Supplementary Material

Gut community alterations associated with *Clostridioides difficile* colonization in hospitalized gastroenterological patients with or without inflammatory bowel disease

Aleksander Mahnic*, Spela Pintar, Pavel Skok, Maja Rupnik

***Corresponding author:**

Aleksander Mahnic aleksander.mahnic@nlzoh.si

PDF includes:

Figures S1 and S2

Tables S1 and S2

OTU	Pearson's coefficient	p Value
Sutterella (Otu00049)	-0.2377	0.0035
Lachnospira (Otu00068)	-0.2279	0.0052
Lachnospiraceae_unclass (Otu00103)	-0.2212	0.0067
Peptostreptococcus (Otu00194)	-0.1992	0.0149
Veillonella (Otu00352)	-0.1912	0.0195
Blautia (Otu00416)	-0.1816	0.0267
Bacteroides (Otu00002)	-0.1809	0.0272
Dialister (Otu00335)	-0.1773	0.0305
Ruminococcus (Otu00113)	-0.1767	0.0311
Veillonella (Otu00038)	-0.1753	0.0324
Lachnospiraceae_unclass (Otu00354)	-0.1727	0.0352
Lachnospiraceae_unclass (Otu00219)	-0.1712	0.0368
Moryella (Otu00481)	-0.1672	0.0415
Klebsiella (Otu00196)	-0.1672	0.0415
Anaerococcus (Otu00454)	-0.1615	0.0491
Lachnospiraceae_unclass (Otu00364)	-0.1614	0.0493
Barnesiella (Otu00040)	0.1636	0.0462
Clostridiales_unclass (Otu00436)	0.1637	0.0460
Ruminococcaceae_unclass (Otu00479)	0.1661	0.0429
Coprococcus (Otu00336)	0.1677	0.0410
Coriobacteriaceae_unclass (Otu00224)	0.1695	0.0387
Clostridiales_unclass (Otu00458)	0.1700	0.0382
Bacteria_unclass (Otu00482)	0.1702	0.0379
Bacteria_unclass (Otu00287)	0.1708	0.0372
Ruminococcaceae_unclass (Otu00134)	0.1712	0.0369
Ruminococcaceae_unclass (Otu00274)	0.1716	0.0364
Ruminococcaceae_unclass (Otu00227)	0.1717	0.0363
Acidaminococcaceae_unclass (Otu00159)	0.1723	0.0357
Lachnospiraceae_unclass (Otu00346)	0.1733	0.0346
Alistipes (Otu00257)	0.1739	0.0339
Romboutsia (Otu00094)	0.1740	0.0338
Clostridium_IV (Otu00472)	0.1780	0.0299
Lachnospiraceae_unclass (Otu00368)	0.1787	0.0292
Ruminococcus (Otu00059)	0.1836	0.0250
Clostridium_IV (Otu00345)	0.1930	0.0183
Desulfovibrionales_unclass (Otu00417)	0.1939	0.0178
Butyrivibrio (Otu00291)	0.1967	0.0162
Firmicutes_unclass (Otu00439)	0.1980	0.0155
Ruminococcaceae_unclass (Otu00321)	0.1985	0.0152
Clostridium_XlVb (Otu00195)	0.1995	0.0147
Parabacteroides (Otu00023)	0.2007	0.0141
Alistipes (Otu00245)	0.2028	0.0131
Ruminococcaceae_unclass (Otu00306)	0.2124	0.0093
Alistipes (Otu00259)	0.2237	0.0061
Ruminococcaceae_unclass (Otu00203)	0.2462	0.0025
Firmicutes_unclass (Otu00135)	0.2466	0.0024
Ruminococcaceae_unclass (Otu00382)	0.2557	0.0017
Firmicutes_unclass (Otu00225)	0.2561	0.0016
Butyricimonas (Otu00164)	0.2591	0.0014
Alistipes (Otu00083)	0.2684	0.0009
Oscillibacter (Otu00013)	0.2844	0.0004
Parabacteroides (Otu00334)	0.3038	0.0002

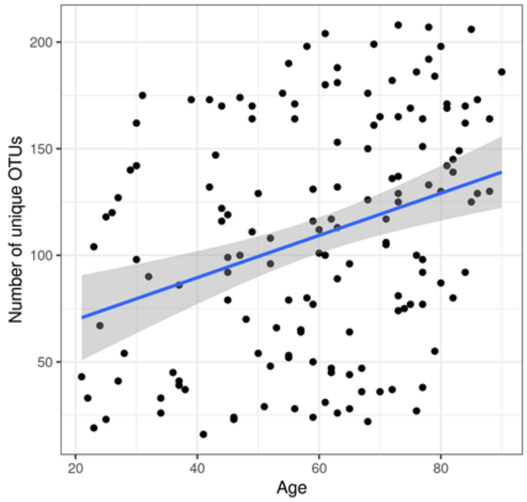

Supplementary Figure S1: Age-associated bacterial community characteristics. Significant correlations (Pearson’s correlation test) between the relative abundances of operational taxonomic units (OTUs) and patient age (left) and the correlation between age and community richness (number of unique OTUs, right).

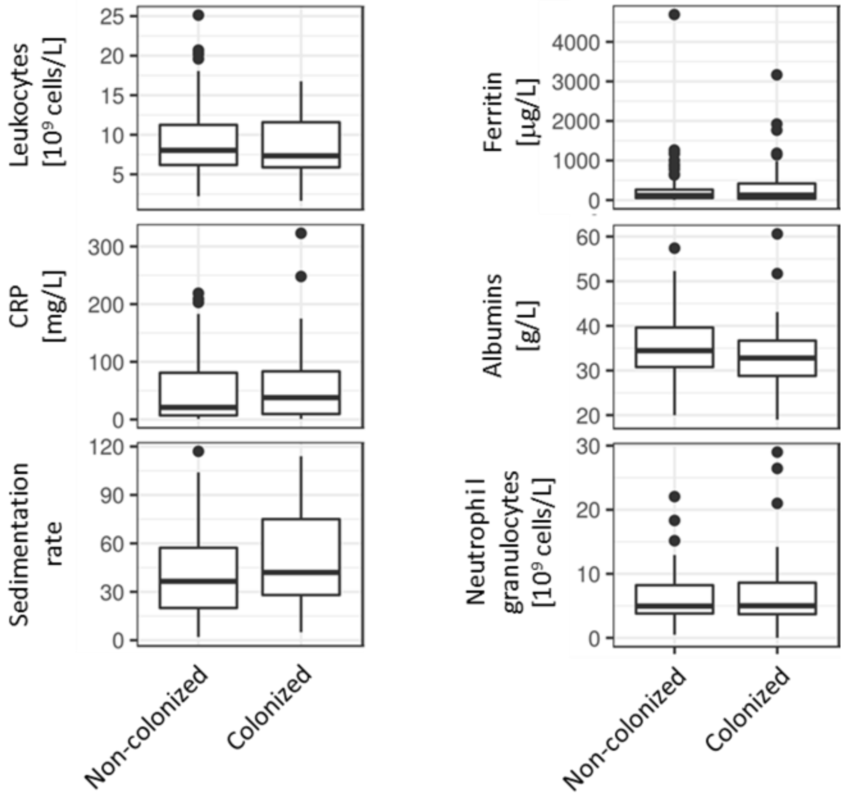

Supplementary Figure S2: Levels of different clinical markers according to *C. difficile* colonization status. Box-plots show levels of inflammatory markers (leukocytes, C-reactive protein (CRP), neutrophil granulocytes, and sedimentation rate), albumins, and ferritin in *C. difficile*-colonized and non-colonized patients. No significant differences were observed.

Supplementary Table S1: Patient metadata and test results for C. difficile-specific qPCR and culture.

Sample	MG-RAST ID	Gender	Age	Diagnosis	Study group	IBD status	Antibiotic At Admission	Antibiotic During Hospitalization	Corticostero-ids Treatment	Biological Treatment	*C. difficile* species-specific qPCR	*tcdB*-specific qPCR	Culture	Culture ribotype
G038	B-G038	Male	21	Crohn's disease	IBD	Remission	Yes	Yes	No	Yes	Negative	Negative	Negative	Negative
G056	B-G056	Male	22	Crohn's disease	IBD	Flare	No	Yes	No	No	31.37	Negative	Negative	Negative
G002	B-G002	Female	23	Crohn's disease	IBD	Flare	No	Yes	No	No	Negative	Negative	Negative	Negative
G008	B-G008	Female	23	Ulcerative colitis	IBD	Flare	No	No	Yes	NA	Negative	Negative	Negative	Negative
G034	B-G034	Female	24	Crohn's disease	IBD	Flare	No	No	No	No	Negative	Negative	Negative	Negative
G053	B-G053	Male	25	Ulcerative colitis	IBD	Flare	No	No	No	No	35.24	Negative	Negative	Negative
G046	B-G046	Female	25	Ulcerative colitis	IBD	Flare	No	Yes	No	No	Negative	Negative	Negative	Negative
G011	B-G011	Male	26	Ulcerative colitis	IBD	Flare	No	No	No	No	Negative	Negative	Negative	Negative
G041	B-G041	Male	27	Other	Non-IBD	NA	No	No	No	No	Negative	Negative	Negative	Negative
G151	B-G151	Female	27	Crohn's disease	IBD	Flare	NA	NA	NA	NA	Negative	Negative	Negative	Negative
G012	B-G012	Male	28	Ulcerative colitis	IBD	Remission	No	No	No	No	37.72	Negative	Negative	Negative
G039	B-G039	Male	29	Ulcerative colitis	IBD	Flare	No	No	No	No	Negative	Negative	Negative	Negative
G147	B-G147	Male	30	Crohn's disease	IBD	Flare	No	Yes	No	No	36.74	Negative	Negative	Negative
G055	B-G055	Male	30	Other	Non-IBD	NA	No	Yes	No	No	Negative	Negative	Negative	Negative
G154	B-G154	Male	30	Infection	Non-IBD	NA	NA	NA	NA	NA	Negative	Negative	Negative	Negative
G042	B-G042	Female	31	Other	Non-IBD	NA	No	No	No	No	Negative	Negative	Negative	Negative
G090	B-G090	Female	32	Other	Non-IBD	NA	No	No	Yes	No	Negative	Negative	Negative	Negative
G083	B-G083	Male	34	Tumour	Non-IBD	NA	No	No	No	No	34.75	Negative	Negative	Negative
G026	B-G026	Female	34	Crohn's disease	IBD	Flare	No	No	No	No	Negative	Negative	Negative	Negative
G035	B-G035	Female	36	Ulcerative colitis	IBD	Flare	No	Yes	No	No	Negative	Negative	Negative	Negative
G060	B-G060	Male	37	Ulcerative colitis	IBD	Flare	No	No	No	No	28.52	Negative	Negative	Negative
G163	B-G163	Female	37	Ulcerative colitis	IBD	Flare	NA	NA	NA	NA	36.05	Negative	Negative	Negative
G009	B-G009	Male	37	Ulcerative colitis	IBD	Flare	No	No	Yes	No	Negative	Negative	Negative	Negative
G097	B-G097	Male	38	Ulcerative colitis	IBD	Flare	No	No	Yes	No	Negative	Negative	Negative	Negative
G006	B-G006	Female	39	Crohn's disease	IBD	Flare	No	No	No	No	Negative	Negative	Negative	Negative
G106	B-G106	Female	41	Infection	Non-IBD	NA	No	Yes	No	No	Negative	Negative	Negative	Negative
G007	B-G007	Female	42	Ulcerative colitis	IBD	Flare	No	No	No	No	Negative	Negative	Negative	Negative
G051	B-G051	Male	42	Other	Non-IBD	NA	No	No	No	No	Negative	Negative	Negative	Negative
G016	B-G016	Male	43	Crohn's disease	IBD	Flare	No	No	Yes	No	Negative	Negative	Negative	Negative
G070	B-G070	Male	44	Crohn's disease	IBD	Flare	No	No	Yes	No	29.82	Negative	Negative	Negative
G003	B-G003	Male	44	Ulcerative colitis	IBD	Flare	No	No	No	No	Negative	Negative	Negative	Negative
G093	B-G093	Male	44	Crohn's disease	IBD	Flare	No	No	No	No	Negative	Negative	Negative	Negative
G067	B-G067	Male	45	Ulcerative colitis	IBD	Flare	No	No	No	Yes	27.91	35.06	Negative	Negative
G066	B-G066	Male	45	Other	Non-IBD	NA	No	No	No	No	30.17	Negative	Negative	Negative
G159	B-G159	Female	45	Infection	Non-IBD	NA	NA	NA	NA	NA	35.29	Negative	Negative	Negative
G160	B-G160	Male	45	Other	Non-IBD	NA	NA	NA	NA	NA	37.28	Negative	Negative	Negative
G013	B-G013	Female	46	Crohn's disease	IBD	Flare	No	Yes	No	Yes	Negative	Negative	Negative	Negative
G136	B-G136	Male	46	Crohn's disease	IBD	Flare	No	Yes	No	No	Negative	Negative	Negative	Negative
G045	B-G045	Male	47	Crohn's disease	IBD	Remission	No	No	No	No	Negative	Negative	Negative	Negative
G138	B-G138	Male	47	Other	Non-IBD	NA	No	No	No	No	Negative	Negative	Negative	Negative
G029	B-G029	Male	48	Infection	Non-IBD	NA	No	Yes	No	No	Negative	Negative	Negative	Negative
G005	B-G005	Female	49	Ulcerative colitis	IBD	Flare	No	Yes	No	Yes	25.42	25.61	Pozitive	R_001/072
G036	B-G036	Female	49	Other	Non-IBD	NA	No	Yes	No	No	Negative	Negative	Negative	Negative
G111	B-G111	Female	49	Infection	Non-IBD	NA	No	No	No	No	Negative	Negative	Negative	Negative
G072	B-G072	Female	50	Ulcerative colitis	IBD	Flare	No	Yes	No	Yes	32.2	Negative	Negative	Negative
G109	B-G109	Male	50	Infection	Non-IBD	NA	No	No	No	Yes	Negative	Negative	Negative	Negative
G153	B-G153	Male	51	Crohn's disease	IBD	Remission	NA	NA	NA	NA	Negative	Negative	Negative	Negative
G084	B-G084	Female	52	Other	Non-IBD	NA	No	Yes	No	No	36.09	Negative	Negative	Negative
G001	B-G001	Female	52	Crohn's disease	IBD	Remission	Yes	Yes	Yes	Yes	Negative	Negative	Negative	Negative
G143	B-G143	Female	52	Other	Non-IBD	NA	No	No	No	No	Negative	Negative	Negative	Negative
G052	B-G052	Male	53	Infection	Non-IBD	NA	Yes	Yes	No	No	37.15	Negative	Negative	Negative
G122	B-G122	Male	54	Tumour	Non-IBD	NA	No	Yes	No	No	Negative	Negative	Negative	Negative
G058	B-G058	Male	55	Ulcerative colitis	IBD	Flare	No	No	Yes	No	37.1	Negative	Negative	Negative
G059	B-G059	Male	55	Ulcerative colitis	IBD	Flare	Yes	Yes	Yes	Yes	34.04	Negative	Negative	Negative
G054	B-G054	Male	55	Ulcerative colitis	IBD	Flare	No	Yes	Yes	No	Negative	Negative	Negative	Negative
G098	B-G098	Male	55	Ulcerative colitis	IBD	Flare	No	Yes	Yes	No	Negative	Negative	Negative	Negative
G082	B-G082	Male	56	Infection	Non-IBD	NA	No	Yes	No	No	33.81	Negative	Negative	Negative
G142	B-G142	Female	56	Other	Non-IBD	NA	No	No	No	No	Negative	Negative	Negative	Negative
G150	B-G150	Female	56	Ulcerative colitis	IBD	Flare	NA	NA	Yes	NA	Negative	Negative	Negative	Negative
G050	B-G050	Female	57	Ulcerative colitis	IBD	Flare	Yes	No	Yes	Yes	38.68	Negative	Negative	Negative
G094	B-G094	Female	57	Other	Non-IBD	NA	No	No	No	No	Negative	Negative	Negative	Negative
G133	B-G133	Female	57	Tumour	Non-IBD	NA	Yes	Yes	No	No	Negative	Negative	Negative	Negative
G074	B-G074	Male	58	Other	Non-IBD	NA	No	Yes	No	No	27.53	32.81	Negative	Negative
G162	B-G162	Male	58	Other	Non-IBD	NA	NA	NA	NA	NA	Negative	Negative	Negative	Negative
G064	B-G064	Female	59	Crohn's disease	IBD	Flare	No	Yes	No	No	31.1	Negative	Negative	Negative
G121	B-G121	Female	59	Other	Non-IBD	NA	No	No	No	No	36.33	Negative	Negative	Negative
G004	B-G004	Female	59	Crohn's disease	IBD	Remission	No	Yes	No	Yes	Negative	Negative	Negative	Negative
G095	B-G095	Male	59	Other	Non-IBD	NA	No	No	No	No	Negative	Negative	Negative	Negative
G123	B-G123	Male	59	Other	Non-IBD	NA	No	No	No	No	Negative	Negative	Negative	Negative
G019	B-G019	Male	60	Infection	Non-IBD	NA	No	Yes	No	No	38.19	Negative	Negative	Negative
G021	B-G021	Male	60	Other	Non-IBD	NA	No	No	No	No	Negative	Negative	Negative	Negative
G146	B-G146	Male	61	Infection	Non-IBD	NA	No	Yes	No	No	36.83	Negative	Negative	Negative
G043	B-G043	Female	61	Ulcerative colitis	IBD	Flare	No	No	No	No	Negative	Negative	Negative	Negative
G088	B-G088	Female	61	Tumour	Non-IBD	NA	No	No	No	No	Negative	Negative	Negative	Negative
G127	B-G127	Male	61	Other	Non-IBD	NA	No	No	No	No	Negative	Negative	Negative	Negative
G115	B-G115	Male	62	Tumour	Non-IBD	NA	No	No	No	No	Negative	Negative	Negative	Negative
G116	B-G116	Female	62	Other	Non-IBD	NA	No	Yes	No	No	Negative	Negative	Negative	Negative
G155	B-G155	Male	62	Other	Non-IBD	NA	NA	NA	NA	NA	Negative	Negative	Negative	Negative
G152	B-G152	Female	63	Ulcerative colitis	IBD	Flare	NA	Yes	NA	NA	38.15	37.85	Pozitive	R_SLO076

G089	B-G089	Male	59	Other	Non-IBD	NA	No	Yes	No	No	34.58	Negative	Negative	Negative
G158	B-G158	Male	63	Infection	Non-IBD	NA	NA	NA	NA	NA	37.38	Negative	Negative	Negative
G010	B-G010	Male	63	Ulcerative colitis	IBD	Flare	No	No	No	No	Negative	Negative	Negative	Negative
G025	B-G025	Male	63	Tumour	Non-IBD	NA	No	Yes	No	No	Negative	Negative	Negative	Negative
G092	B-G092	Female	63	Ulcerative colitis	IBD	Flare	No	Yes	No	No	Negative	Negative	Negative	Negative
G112	B-G112	Female	63	Other	Non-IBD	NA	No	No	No	No	Negative	Negative	Negative	Negative
G076	B-G076	Female	65	Infection	Non-IBD	NA	No	Yes	No	No	31.71	Negative	Negative	Negative
G080	B-G080	Female	65	Infection	Non-IBD	NA	No	Yes	No	No	36.95	Negative	Negative	Negative
G081	B-G081	Female	65	Infection	Non-IBD	NA	No	No	No	No	35.99	Negative	Negative	Negative
G113	B-G113	Female	65	Infection	Non-IBD	NA	Yes	Yes	No	No	Negative	Negative	Negative	Negative
G037	B-G037	Female	67	Crohn's disease	IBD	Flare	No	Yes	No	No	Negative	Negative	Negative	Negative
G108	B-G108	Female	67	Other	Non-IBD	NA	No	No	No	No	Negative	Negative	Negative	Negative
G015	B-G015	Female	68	Crohn's disease	IBD	Flare	No	Yes	Yes	NA	Negative	Negative	Negative	Negative
G017	B-G017	Male	68	Infection	Non-IBD	NA	No	Yes	No	No	Negative	Negative	Negative	Negative
G022	B-G022	Male	68	Infection	Non-IBD	NA	No	No	No	No	Negative	Negative	Negative	Negative
G130	B-G130	Female	68	Tumour	Non-IBD	NA	No	No	No	No	Negative	Negative	Negative	Negative
G086	B-G086	Male	69	Tumour	Non-IBD	NA	No	No	No	No	Negative	Negative	Negative	Negative
G096	B-G096	Male	69	Infection	Non-IBD	NA	No	No	No	No	Negative	Negative	Negative	Negative
G057	B-G057	Male	70	Other	Non-IBD	NA	No	No	No	No	36.62	Negative	Negative	Negative
G124	B-G124	Male	70	Infection	Non-IBD	NA	No	Yes	No	No	Negative	Negative	Negative	Negative
G065	B-G065	Male	71	Infection	Non-IBD	NA	No	No	Yes	No	30.32	37.16	Negative	Negative
G077	B-G077	Female	71	Infection	Non-IBD	NA	No	Yes	No	No	31.94	Negative	Negative	Negative
G031	B-G031	Female	71	Tumour	Non-IBD	NA	Yes	Yes	No	No	Negative	Negative	Negative	Negative
G101	B-G101	Male	72	Other	Non-IBD	NA	No	No	No	No	23.85	Negative	Pozitive	R_027
G087	B-G087	Male	72	Tumour	Non-IBD	NA	No	Yes	No	No	Negative	Negative	Negative	Negative
G148	B-G148	Male	72	Tumour	Non-IBD	NA	No	No	No	No	Negative	Negative	Negative	Negative
G129	B-G129	Female	73	Other	Non-IBD	NA	No	No	No	No	37.61	Negative	Negative	Negative
G048	B-G048	Female	73	Other	Non-IBD	NA	No	Yes	No	No	Negative	Negative	Negative	Negative
G079	B-G079	Female	73	Other	Non-IBD	NA	No	No	No	No	Negative	Negative	Negative	Negative
G085	B-G085	Female	73	Other	Non-IBD	NA	Yes	Yes	No	No	Negative	Negative	Negative	Negative
G126	B-G126	Male	73	Tumour	Non-IBD	NA	No	No	No	No	Negative	Negative	Negative	Negative
G134	B-G134	Female	73	Other	Non-IBD	NA	No	Yes	No	No	Negative	Negative	Negative	Negative
G137	B-G137	Female	73	Tumour	Non-IBD	NA	No	No	No	No	Negative	Negative	Negative	Negative
G161	B-G161	Female	74	Other	Non-IBD	NA	NA	NA	NA	NA	Negative	Negative	Negative	Negative
G135	B-G135	Male	75	Tumour	Non-IBD	NA	No	Yes	No	No	27.52	31.27	Pozitive	R_002
G030	B-G030	Male	75	Tumour	Non-IBD	NA	No	No	No	No	Negative	Negative	Negative	Negative
G078	B-G078	Male	76	Other	Non-IBD	NA	No	No	No	No	34.36	Negative	Negative	Negative
G120	B-G120	Male	76	Tumour	Non-IBD	NA	No	No	No	No	37.71	Negative	Negative	Negative
G018	B-G018	Male	76	Infection	Non-IBD	NA	No	Yes	No	No	Negative	Negative	Negative	Negative
G062	B-G062	Female	77	Infection	Non-IBD	NA	No	Yes	No	No	28.85	35.75	Negative	Negative
G061	B-G061	Female	77	Ulcerative colitis	IBD	Flare	No	No	No	No	31.26	Negative	Negative	Negative
G118	B-G118	Male	77	Tumour	Non-IBD	NA	No	No	No	No	37.03	Negative	Negative	Negative
G033	B-G033	Female	77	Infection	Non-IBD	NA	No	Yes	No	No	Negative	Negative	Negative	Negative
G131	B-G131	Female	77	Other	Non-IBD	NA	No	No	No	No	Negative	Negative	Negative	Negative
G157	B-G157	Female	77	Tumour	Non-IBD	NA	NA	NA	NA	NA	Negative	Negative	Negative	Negative
G047	B-G047	Female	78	Other	Non-IBD	NA	Yes	Yes	No	No	Negative	Negative	Negative	Negative
G117	B-G117	Male	78	Tumour	Non-IBD	NA	No	No	No	No	Negative	Negative	Negative	Negative
G149	B-G149	Female	78	Other	Non-IBD	NA	No	No	No	No	Negative	Negative	Negative	Negative
G024	B-G024	Female	79	Tumour	Non-IBD	NA	No	No	No	No	35.04	Negative	Negative	Negative
G023	B-G023	Female	79	Tumour	Non-IBD	NA	No	No	No	No	Negative	Negative	Negative	Negative
G027	B-G027	Female	80	Infection	Non-IBD	NA	No	No	No	No	Negative	Negative	Negative	Negative
G049	B-G049	Female	80	Infection	Non-IBD	NA	No	Yes	No	No	Negative	Negative	Negative	Negative
G110	B-G110	Female	80	Tumour	Non-IBD	NA	No	No	No	No	Negative	Negative	Negative	Negative
G099	B-G099	Male	81	Other	Non-IBD	NA	No	No	No	No	Negative	Negative	Negative	Negative
G107	B-G107	Female	81	Other	Non-IBD	NA	No	No	No	No	Negative	Negative	Negative	Negative
G144	B-G144	Female	81	Other	Non-IBD	NA	No	No	No	No	Negative	Negative	Negative	Negative
G075	B-G075	Male	82	Other	Non-IBD	NA	No	No	No	No	33.83	Negative	Negative	Negative
G028	B-G028	Female	82	Infection	Non-IBD	NA	No	Yes	No	No	Negative	Negative	Negative	Negative
G156	B-G156	Male	82	Tumour	Non-IBD	NA	NA	NA	NA	NA	Negative	Negative	Negative	Negative
G063	B-G063	Male	83	Infection	Non-IBD	NA	No	No	No	No	32.04	Negative	Negative	Negative
G020	B-G020	Female	84	Tumour	Non-IBD	NA	No	Yes	No	No	35.75	Negative	Pozitive	R_002
G073	B-G073	Female	84	Other	Non-IBD	NA	No	No	No	No	30.82	Negative	Negative	Negative
G032	B-G032	Male	84	Tumour	Non-IBD	NA	No	Yes	No	No	Negative	Negative	Negative	Negative
G103	B-G103	Female	85	Other	Non-IBD	NA	No	No	No	No	Negative	Negative	Negative	Negative
G125	B-G125	Male	85	Tumour	Non-IBD	NA	No	No	No	No	Negative	Negative	Negative	Negative
G068	B-G068	Female	86	Infection	Non-IBD	NA	No	Yes	No	No	31.05	Negative	Negative	Negative
G132	B-G132	Female	86	Other	Non-IBD	NA	No	Yes	No	No	Negative	Negative	Negative	Negative
G091	B-G091	Female	88	Ulcerative colitis	IBD	Remission	No	No	No	No	Negative	Negative	Negative	Negative
G100	B-G100	Female	88	Other	Non-IBD	NA	No	No	No	No	Negative	Negative	Negative	Negative
G140	B-G140	Female	90	Other	Non-IBD	NA	No	No	No	No	Negative	Negative	Negative	Negative

Supplementary Table S2: Permutational multivariate analysis of variance between bacterial community and four host-specific factors.

	R2	pValue	
**Gender**	0.008	0.22577	
**Age**	0.0147	0.00599	**
**Diagnosis**	0.0106	0.03896	*
***C.difficile* colonization**	0.0115	0.02098	*
